# Jacob's Syndrome and Hearing Loss: A Case Study

**DOI:** 10.1002/ccr3.72143

**Published:** 2026-02-25

**Authors:** Houra Bagheri, Ali Kouhi, Mojtaba Alidoust, Amineh Koravand

**Affiliations:** ^1^ Department of Audiology, School of Rehabilitation Shahid Beheshti University of Medical Science Tehran Iran; ^2^ Associate Professor and Faculty Member of the Department of Otolaryngology, Head and Neck Surgery Tehran University of Medical Sciences Tehran Iran; ^3^ Associate Professor, Audiology and Speech‐Language Pathology Program, School of Rehabilitation Sciences, Faculty of Health Sciences University of Ottawa Ottawa Canada

**Keywords:** audiology, conductive hearing loss, jacob syndrome, otology, otosclerosis

## Abstract

Jacob's syndrome, or XYY syndrome, is caused by the presence of an extra Y chromosome in each male human cell. Although this extra Y chromosome makes these people taller than the average, they do not show any other unusual physical characteristics. Until now, very few studies have specifically examined the hearing abnormalities of individuals with this syndrome. A four‐year‐old boy with Jacob's syndrome was referred for an auditory assessment due to speech delay. The test battery results revealed a conductive hearing loss bilaterally based on existing air–bone gaps in pure‐tone audiometry, absence of distortion product otoacoustic emissions, and stapedial reflexes. Furthermore, the auditory brainstem responses confirmed a conductive hearing loss, as the interwave interval between waves one, three, and five was normal. Still, the absolute latency of all waves was delayed. These results were consistent with the CT (Computed Tomography) scan images demonstrating stapes footplate fixation. The audiological and imaging findings were supportive of stapes fixation; however, they were not sufficient to establish a definitive etiological diagnosis. Bilateral stapes fixation was suspected in a young boy with Jacob's syndrome. Behavioral, physiological, and electrophysiological hearing assessments indicated mild bilateral conductive hearing loss, with findings that were consistent with and supported by medical imaging. Although the exact etiology of the stapes fixation could not be determined, both congenital and acquired causes were considered. Incorporating comprehensive audiological and otological evaluations into the routine follow‐up of children with Jacob's syndrome may facilitate early identification of hearing impairment, particularly during critical periods of speech and language development.

## Summary

Early audiological and otological assessment is essential in children with Jacob's syndrome. This case highlights the importance of evaluating conductive hearing loss in syndromic patients, including consideration of stapes fixation as a potential etiology. Early identification of hearing impairment ensures timely management and supports optimal speech and language development outcomes.

## Graphical abstract

Bilateral mild‐to‐moderate hearing loss identified in a child with Jacob’s syndrome (47,XYY). This case highlights the importance of early audiological assessment in children with chromosomal abnormalities to facilitate timely intervention and optimize developmental outcomes.

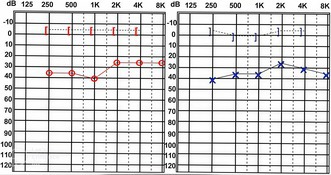

## 1 Introduction

Jacob's syndrome, also known as 47, XYY syndrome, is a rare genetic condition affecting male children [1]. It is categorized under “sex chromosome trisomies” [2], of which Klinefelter's syndrome is the most common [3, 4]. This syndrome was first described by Sandberg et al. in 1961 and Jacobs et al. in 1965 [1, 5] and is estimated to affect about 1 in 1000 male children [6]. Evidence shows that spermatogonia and/or spermatocytes with an additional Y chromosome are selectively excluded during gametogenesis, and the 47, XYY genotype is most probably a result of nondisjunction during meiotic division (MII) during spermatogenesis or postzygotic mitosis [6, 7, 8, 9].

Jacob's syndrome is associated with various physical, neurological, and developmental characteristics. Patients with this condition may experience sluggish motor activities, excessive acne, multiple joint problems, and neurodevelopmental disorders such as language difficulties, social–emotional challenges, autism spectrum disorder (ASD), and attention deficit hyperactivity disorder (ADHD) [10, 11, 12, 13]. Additionally, developmental anomalies, including abnormalities in the palatal and mandibular arch, skeletal structure, and vertebrae, have been linked to this trisomy [14, 15, 16].

Some of the most common physical traits observed in individuals with Jacob's syndrome include tall stature, brachydactyly (short fingers), pes planus (flat feet), and hypotonia (reduced muscle tone) [17]. In dental examinations, affected children frequently present with conditions such as open bite, macrodontia (abnormally large teeth), or taurodontia (enlarged pulp chambers in molars) [10].

Beyond physical characteristics, some studies have explored psychological and behavioral aspects associated with Jacob's syndrome [18, 19]. Jacobs et al. (1965) [1] identified seven cases of XYY chromosome configuration among 197 individuals with violent or criminal tendencies admitted to the Scottish State Hospital in Edinburgh [1]. However, psychological tendencies were not the only focus of research. Another study found that three out of thirty‐four prisoners over 175 cm in height had Jacob's syndrome, suggesting a potential correlation with stature [20].

Despite the cognitive and language disorders as well as the physical abnormalities in the head, only a few studies have investigated the possible effects of this syndrome on hearing in children, which we mention. In one study, evoked auditory potential latencies were compared in children with autism spectrum disorder (ASD), typically developing children, and XYY boys [21]. This study highlights the impact of 47, XYY syndrome on auditory evoked response latencies, particularly the M50 and M100 components. M50 and M100 are auditory evoked responses measurable by Magnetoencephalography. M100 peaks ~100 ms poststimulus and reflects early cortical processing of sound features. Though less studied, M50 (~50 ms) is considered a middle‐latency response more prominent in children and linked to early sensory encoding [22]. Findings indicate a left hemisphere delay in XYY (M50: 7.9 ms), contrasting with the right hemisphere delay in ASD (M50: 5.6 ms), suggesting an asymmetric role of Y chromosome genes in auditory processing. Findings related to the later M100 component showed a similar trend but did not reach statistical significance due to higher variability. While previous studies linked delayed auditory responses to abnormal neural maturation, this study emphasizes distinct hemispheric effects in XYY and ASD, suggesting further investigation. This paper had no data about the behavioral tests [21].

In the current study, the hearing condition of a child with Jacob's syndrome was examined by using a combination of behavioral, electrophysiological, and physiological tests.

## 2 Case History

**FIGURE 1:**
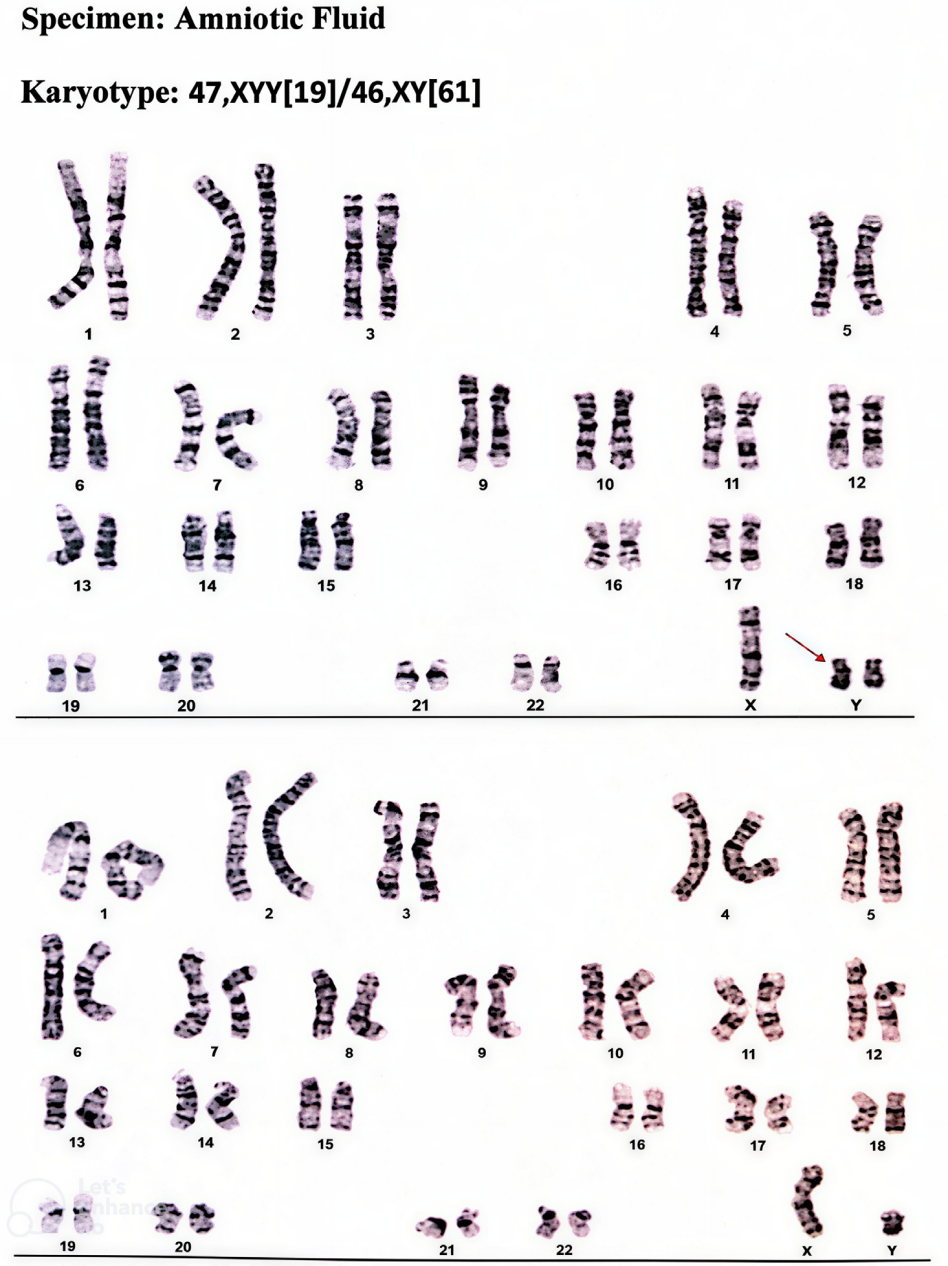
The karyotype test result. The extra Y chromosome is shown by the red arrow.

**FIGURE 2:**
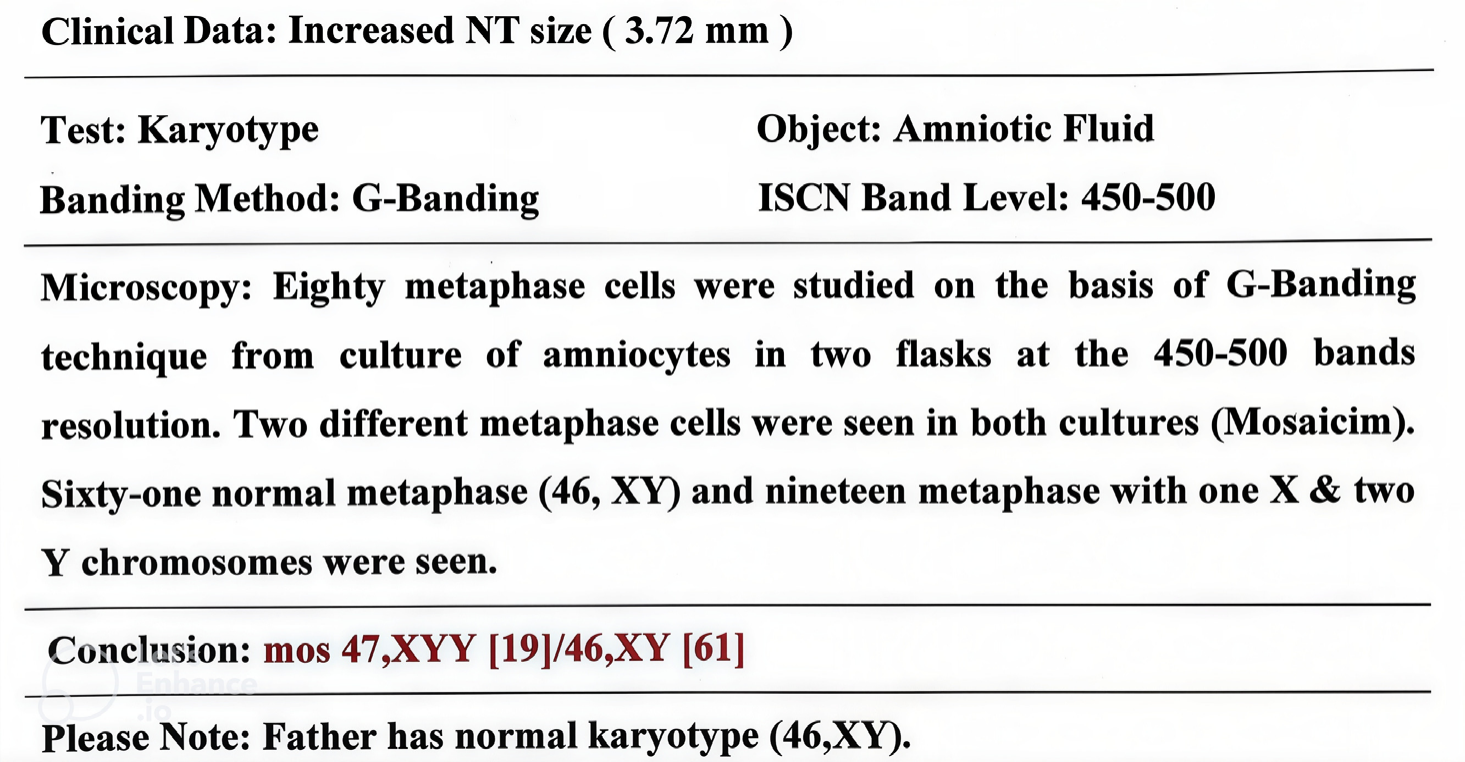
The karyotype test report.

A four‐year‐old child with Jacob's syndrome was referred to the audiology clinic of Mofid Children's Hospital by a pediatric neurologist due to speech delay. The child's mother was 21 years old at the time of the child's delivery. Natural delivery occurred at 41 weeks of gestation. Jacob's syndrome diagnosis was suspected due to increased nuchal translucency (NT) thickness, which was reported to be 3.72 mm at the age of 16 weeks +1 day. The presence of fetal nuchal translucency > 3 mm during the first trimester is a useful indicator of fetal chromosomal abnormalities [23]. Further investigations included a referral for amniocentesis, and results showed a chromosomal abnormality. Eighty metaphase cells were studied based on the G‐Banding technique from a culture of amniocytes in two flasks at the 450–500 band resolution. Two different metaphase cells were seen in both cultures (mosaicism). Sixty‐one normal metaphases (46, XY) and 19 metaphases with one X and two Y chromosomes were seen (Figures 1 and 2). Jacob's syndrome was thus confirmed during the 12th fetal week. None of the other family members had this syndrome.

During the early months, the child experienced malnutrition and a swallowing disorder, which was diagnosed as a weakness in the throat muscles by a speech therapist. He received specific rehabilitation for both issues. By around age 3, behavioral difficulties such as obsessive tendencies and communication challenges like poor listening and speaking abilities, along with difficulty participating in group activities with peers, were also observed and confirmed by a pediatric neurologist. The malnutrition and swallowing disorder improved after 10 months of therapy, which included a combination of speech therapy and occupational therapy. The child's therapy is still ongoing, aiming to address any remaining issues.

## 3 Methods

### 3.1 Differential Diagnosis

A differential diagnostic approach was undertaken to identify the presence and nature of any hearing impairment in the child. Both conductive and sensorineural etiologies were considered, as well as possible middle ear and inner ear pathologies. Clinical observations and audiological findings guided the selection of further diagnostic procedures to clarify the underlying condition.

### 3.2 Investigations

Audiological examinations were conducted using both behavioral and neurophysiological measures. The test battery included play audiometry, tympanometry, acoustic reflex testing, distortion product otoacoustic emissions (DPOAE), and auditory brainstem response (ABR). Audiometry was performed using a GSI 61 Clinical Audiometer. Tympanometry and acoustic reflex tests were conducted with a GSI Tympstar device. DPOAE recording and ABR threshold tracing were performed using the AUDERA system (GSI, Eden Prairie, MN).

Audiometry, DPOAE, and ABR tests were carried out in a soundproof audiometric booth. During electrophysiological testing, the child was sedated with chloral hydrate syrup prescribed by a pediatrician. Given the high level of cooperation demonstrated by the child and the reliability of results obtained across multiple behavioral test sessions, air conduction (AC) and bone conduction (BC) audiometry thresholds were considered reliable and used for interpretation.

Play audiometry was conducted to measure hearing thresholds across frequencies ranging from 250 to 8000 Hz. At the beginning of the assessment, a warble tone was used to familiarize the child with the test procedure, followed by pure‐tone stimuli to determine hearing thresholds. Ear‐specific measurements were obtained using circumaural headphones, allowing each ear to be assessed separately.

Tympanometry and acoustic reflex testing were performed in a quiet environment while ensuring that the child remained still and did not speak.

DPOAE testing covered frequency ranges from 250 to 8000 Hz. Signal‐to‐noise ratio (SNR) pass criteria were defined as a minimum of 3 dB across all four tested frequencies or at least 5 dB in three out of four frequencies screened [24, 25, 26].

The AC‐ABR testing was performed using alternating click stimuli with a duration of 0.1 milliseconds, delivered via earphones. An ipsilateral electrode montage was used, with electrode impedance maintained below 5 kΩ and interelectrode impedance below 2 kΩ. The stimulus intensity began at 60 dB nHL, decreased in 20 dB steps, and was then increased in 10 or 5 dB increments until Wave V was no longer detectable. The ABR threshold was defined as the lowest intensity level at which Wave V could be reliably identified. To further evaluate wave morphology, the stimulus intensity was increased to 80 dB nHL to assess the presence of early waves (Waves I and III).

To investigate the etiology of the conductive hearing loss, a high‐resolution temporal bone CT scan prescribed by an Ear, Nose, and Throat (ENT) Specialist was performed.

### 3.3 Treatment

At this stage, no therapeutic intervention was initiated. The primary objective was to establish an accurate diagnosis through comprehensive audiological and radiological evaluation. Management decisions were deferred pending confirmation of the diagnostic findings.

## 4 Results

**FIGURE 3:**
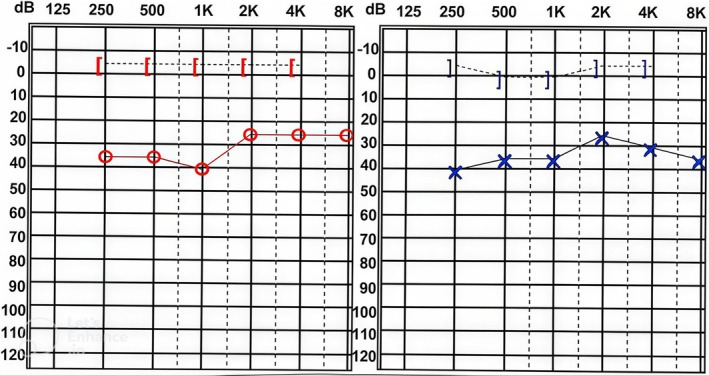
The behavioral audiometry results. × = air conduction (unmasked) left ear; ] = bone conduction (masked) left ear; ○ = air conduction (unmasked) right ear; [ = bone conduction (masked) right ear.

Play audiometry revealed mild conductive hearing loss in air conduction thresholds across frequencies ranging from 250 to 8000 Hz in both ears (Figure 3). Bone conduction thresholds were within normal limits, indicating the presence of an air–bone gap.

Given that the primary reason for referral was a speech disorder, speech audiometry was not performed during the diagnostic process.

**FIGURE 4:**
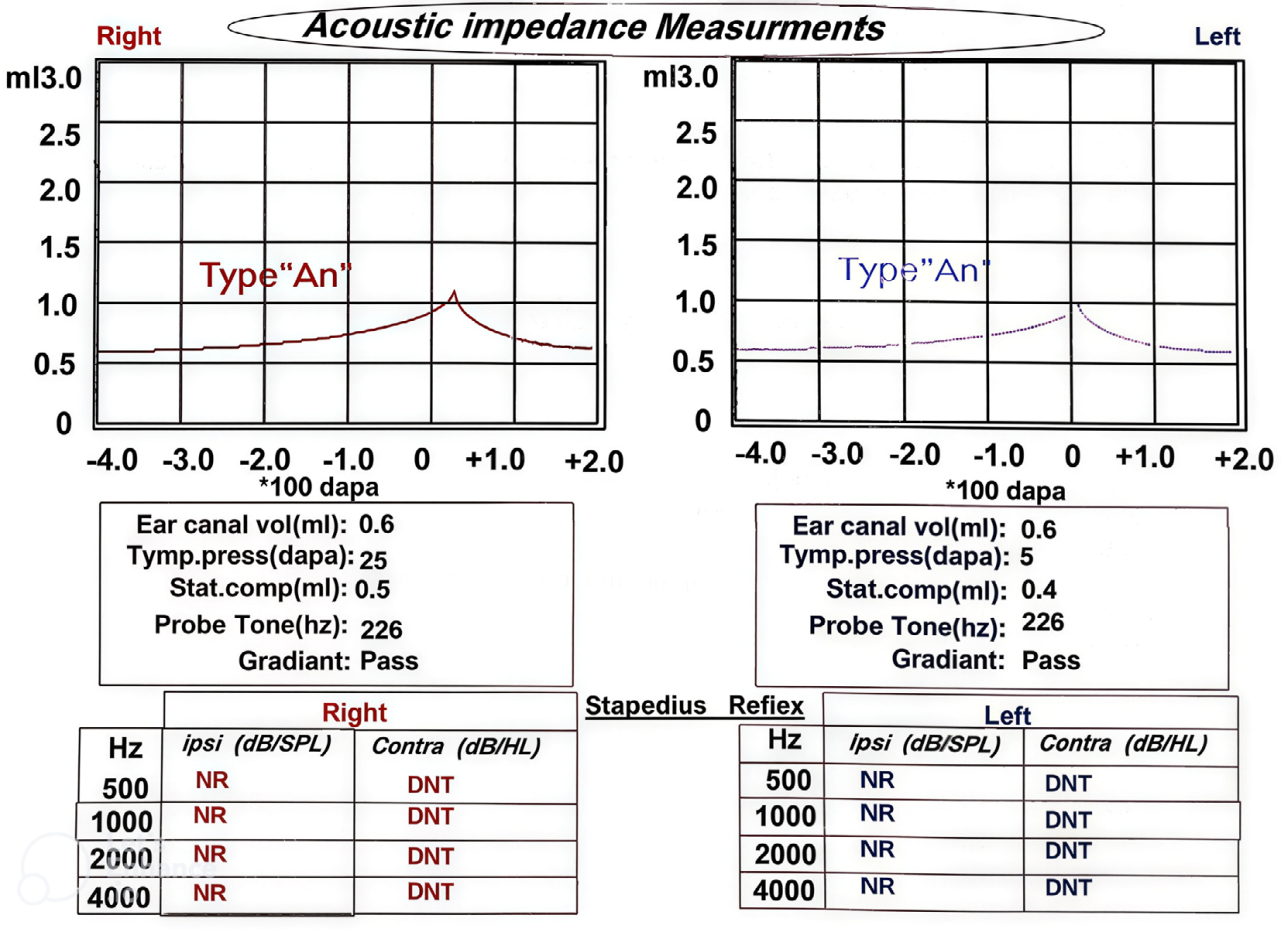
The Tympanometry and Acoustic Reflex test results. Normal middle ear function was seen, but acoustic reflexes were absent.

Tympanometric findings suggested normal tympanic membrane and middle ear function bilaterally, with Type A tympanograms observed in both ears. Ear canal volume was measured at 0.6 mL bilaterally. Tympanic pressure values were 25 daPa in the right ear and 5 daPa in the left ear, while static compliance values were 0.5 and 0.4 mL for the right and left ears, respectively (Figure 4). Ipsilateral acoustic reflexes were absent in both ears at all tested frequencies (500, 1000, 2000, and 4000 Hz) (Figure 4). Contralateral acoustic reflexes were not assessed.

**FIGURE 5:**
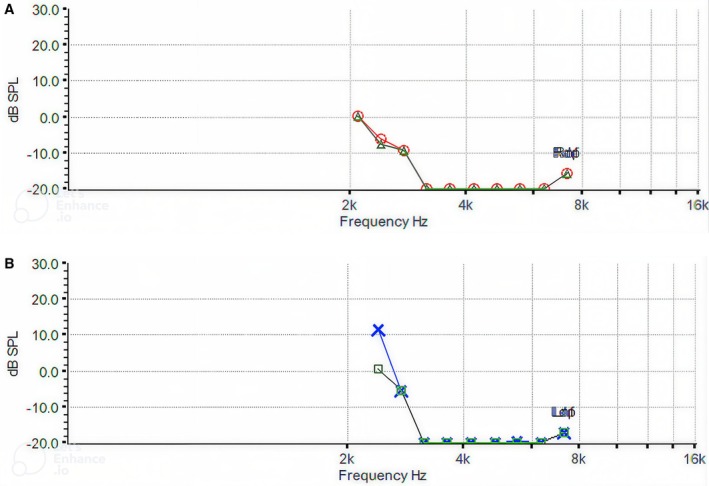
(A) Right ear DPOAE. (B) Left ear DPOAE.

DPOAE responses were absent bilaterally in the frequency range from 2000 to 8000 Hz (Figure 5).

**FIGURE 6:**
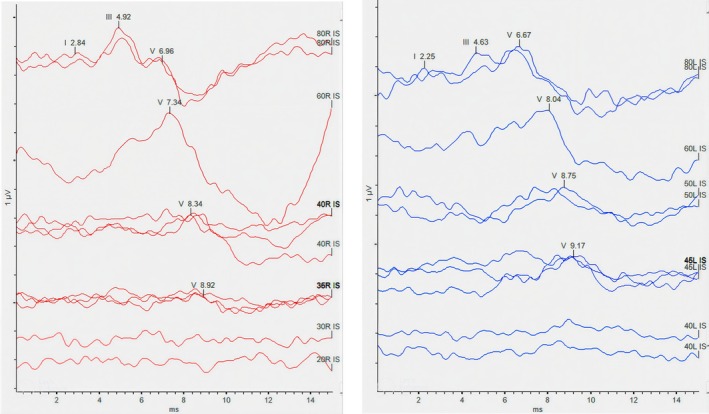
Auditory brainstem response results. In the right side (red), the threshold to 35 dB nHL was tracked, and in the left side (blue), the threshold to 45 dB nHL was tracked.

ABR testing demonstrated delayed absolute latencies of all waves, with preserved interwave intervals, a pattern consistent with conductive hearing loss. Poor morphology or absence of early waves (Waves I and III) was observed even at higher intensity levels, while Wave V remained identifiable. ABR threshold estimation revealed thresholds of 35 dB nHL in the right ear and 45 dB nHL in the left ear (Figure 6).

**FIGURE 7:**
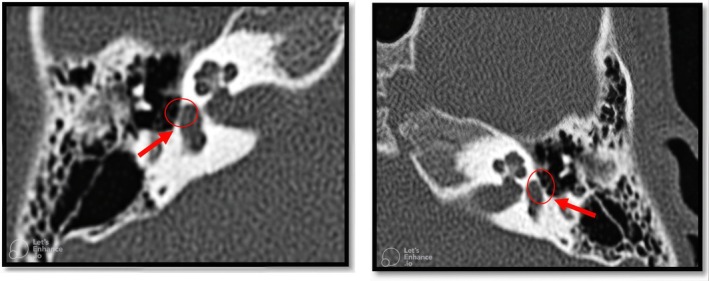
The CT scan images. The location of the footplate is marked in both images from different angles, which is a little thicker in these pictures that increase the footplate fixation probability.

Temporal bone CT imaging demonstrated a normal middle ear cavity and ossicular chain. However, fixation of the stapes footplate was identified. The footplate thickness was marginal, and no evidence of spongiosis was observed in the fissure ante fenestram (Figure 7).

Overall, behavioral, physiological, and electrophysiological auditory assessments indicated mild bilateral conductive hearing loss in this child with Jacob's syndrome. The presence of an air–bone gap on behavioral audiometry, along with normal tympanometric findings and absent acoustic reflexes, supported the diagnosis of conductive hearing loss. Additional supportive evidence included absent DPOAE responses, delayed ABR wave latencies with preserved interwave intervals, and imaging findings consistent with stapes footplate fixation.

## 5 Discussion

Jacob's syndrome, also known as 47, XYY syndrome, is a genetic disorder characterized by the presence of an additional Y chromosome in males [1]. Although the physical and developmental features of this syndrome have been widely investigated, its potential impact on hearing health has received comparatively limited attention. The presence of conductive hearing loss in the current case highlights the importance of comprehensive audiological assessment in individuals with Jacob's syndrome.

Conductive hearing loss results from abnormalities affecting the outer or middle ear, leading to impaired sound transmission to the inner ear [27]. In the present case, conductive hearing loss was identified through a combination of behavioral, physiological, and electrophysiological assessments. Behavioral audiometry demonstrated a conductive pattern, accompanied by absent acoustic reflexes and delayed absolute wave latencies on auditory brainstem response testing, with preserved interwave intervals. Together, these findings are consistent with a conductive impairment and are compatible with stapes fixation rather than providing definitive evidence for a specific underlying etiology.

Stapes fixation in pediatric patients may occur either due to congenital fixation or gradual otospongiosis origins, and distinguishing between these etiologies can be challenging, particularly in the absence of definitive radiological findings. Although otosclerosis represents one possible cause of stapes fixation, it is typically diagnosed later in life and is relatively uncommon in young children [28]. In this case, temporal bone CT imaging demonstrated stapes footplate fixation without evidence of spongiosis at the fissure ante fenestram, a finding that does not exclude otosclerosis but also does not confirm it. Therefore, the use of the broader term stapes fixation was considered more appropriate to reflect the diagnostic uncertainty.

The underlying cause of stapes fixation in individuals with Jacob's syndrome remains unclear. Genetic factors may play a role, as both otosclerosis and other middle ear abnormalities have been associated with genetic influences and familial clustering [29, 30, 31]. In addition, developmental abnormalities reported in Jacob's syndrome, including craniofacial and skeletal variations [14, 15, 16], may predispose affected individuals to otological involvement. Given the relatively high prevalence of skeletal and bone‐related abnormalities in this syndrome, further investigation into possible associations with middle ear ossicular dysfunction appears warranted.

Hormonal factors may also be relevant. Elevated testosterone levels associated with the presence of an additional Y chromosome [10] have been proposed to influence bone metabolism, although their role in middle ear pathology remains speculative. Notably, otosclerosis is more frequently reported in adults than in children [28] and is more prevalent in females than males [30, 32]. Consequently, the occurrence of stapes fixation compatible with otosclerosis‐related pathology in a young male child with Jacob's syndrome represents an uncommon clinical presentation.

Overall, the identification of conductive hearing loss in this patient underscores the importance of early recognition and intervention in children with Jacob's syndrome. Untreated conductive hearing loss can adversely affect speech and language development, social interaction, and academic performance, particularly during early childhood [33]. Early referral for audiological evaluation and appropriate management strategies may therefore play a critical role in optimizing developmental outcomes.

One limitation of this case relates to the uncertainty surrounding the exact etiology of the stapes fixation. The interpretation was based on a combination of clinical findings, audiological assessments, and radiological imaging, all of which were supportive but not conclusive. As a result, it was not possible to clearly distinguish between congenital stapes fixation and early‐onset otosclerosis in this patient.

This case further emphasizes the value of a multidisciplinary approach in the management of genetic conditions such as Jacob's syndrome. Collaboration among pediatricians, geneticists, neurologists, audiologists, and otolaryngologists is essential to ensure comprehensive assessment and to address the diverse medical and developmental needs of affected children and their families.

## 6 Conclusion

In conclusion, this case report contributes to the limited literature addressing potential otological manifestations of Jacob's syndrome and highlights the importance of considering hearing health in the clinical management of individuals with genetic disorders. The findings suggest the presence of stapes fixation associated with mild bilateral conductive hearing loss; however, the precise etiology could not be determined.

Middle ear abnormalities, including stapes fixation potentially related to otosclerosis or other mechanisms, may represent one aspect of the clinical spectrum of Jacob's syndrome. At the same time, the increased prevalence of communication and neurodevelopmental difficulties, such as autism spectrum features, reported in this population [34], may complicate the recognition of hearing impairment when communication challenges are present.

Although a direct causal relationship between Jacob's syndrome and stapes fixation cannot be established based on this single case, periodic audiological and otological evaluations during critical periods of speech and language development may facilitate earlier detection of hearing disorders. Further studies are needed to investigate the prevalence, characteristics, and clinical significance of middle ear involvement in children with Jacob's syndrome, which may help inform future screening and management recommendations.

## Author Contributions

**Houra Bagheri:** conceptualization, data curation, funding acquisition, investigation, project administration, resources, visualization, writing – original draft, writing – review and editing. **Ali Kouhi:** investigation, resources, validation, writing – original draft, writing – review and editing. **Mojtaba Alidoust:** methodology, visualization, writing – review and editing. **Amineh Koravand:** supervision, validation, writing – review and editing.

## Funding

The authors have nothing to report.

## Consent

Written informed consent was obtained from the patient's parents/legal guardians before submission of this case report. The parents/legal guardians have been fully informed about the publication of clinical data and images, understand that this information will be published on an open‐access basis, and have agreed to the use and dissemination of the patient's information. The authors retain a copy of the signed consent form and will provide it to the publisher upon request.

## Data Availability

The data that support the findings of this study are available on request from the corresponding author. The data are not publicly available due to privacy or ethical restrictions.
